# Understanding the tolerance of halophilic archaea to stress landscapes

**DOI:** 10.1111/1758-2229.70039

**Published:** 2024-11-20

**Authors:** Laura Matarredona, Basilio Zafrilla, Mónica Camacho, María‐José Bonete, Julia Esclapez

**Affiliations:** ^1^ Department of Biochemistry and Molecular Biology and Soil Science and Agricultural Chemistry, Faculty of Science University of Alicante Alicante Spain

## Abstract

Haloarchaea, known for their resilience to environmental fluctuations, require a minimum salt concentration of 10% (w/v) for growth and can survive up to 35% (w/v) salinity. In biotechnology, these halophiles have diverse industrial applications. This study investigates the tolerance responses of nine haloarchaea: *Haloferax mediterranei*, *Haloferax volcanii*, *Haloferax gibbonsii*, *Halorubrum californiense*, *Halorubrum litoreum, Natrinema pellirubrum*, *Natrinema altunense*, *Haloterrigena thermotolerans* and *Haloarcula sinaiiensis*, under various stressful conditions. All these archaea demonstrated the ability to thrive in the presence of toxic metals such as chromium, nickel, cobalt and arsenic, and their tolerance to significantly elevated lithium concentrations in the medium was remarkable. Among the studied haloarchaea, *Hfx. mediterranei* exhibited superior resilience, particularly against lithium, with an impressive minimum inhibitory concentration (MIC) of up to 4 M LiCl, even replacing NaCl entirely. *Haloferax* species showed specificity for conditions with maximal growth rates, while *Htg. thermotolerans* and *Nnm. altunense* displayed high resilience without losing growth throughout the ranges, although these were generally low. ICP‐MS results highlighted the impressive intracellular lithium accumulation in *Nnm. pellirubrum*, emphasizing its potential significance in bioremediation. This research highlights a new characteristic of haloarchaea, their tolerance to high lithium concentrations and the potential for new applications in extreme industrial processes and bioremediation.

## Graphical abstract

The tolerance responses of nine Haloarchaea species were analysed under 70 different conditions, including various salinities, temperatures, oxidative stress and the presence of metals. In general, all characterised species tolerated heavy metals, with a particularly striking tolerance to lithium. These results highlight the relevance of Haloarchaea in bioremediation efforts as an environmentally friendly biological methodology, especially *Haloferax mediterranei* and *Natrinema pellirubrum.*

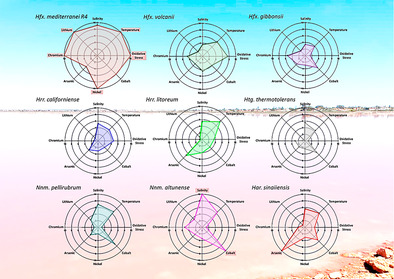

## INTRODUCTION

Microorganisms inhabiting saline environments have undergone molecular adaptations to maintain cellular homeostasis and integrity in response to fluctuating and severe circumstances, such as desiccation, intense irradiation or rapid salinity changes. As a result, haloarchaea have evolved with remarkable adaptability across a broad spectrum of temperatures (4–50°C), pH levels (4–10) and extreme levels of oxidative stress (Bowers & Wiegel, 2011; Dawson et al., 2012; Franzmann et al., 1988; Matarredona et al., 2021; Mullakhanbhai & Larsen, 1975; Shahmohammadi et al., 1998). However, the behaviour of haloarchaea under other stressors, such as heavy metals and hydrogen peroxide, still needs more in‐depth studies.

The escalating impact of climate change and increased pollution from anthropogenic activities and industrialization are causing an increase in water salinity. Consequently, using moderate or extreme halophilic microorganisms with bioremediation capabilities becomes advantageous over other mesophilic microorganisms, commonly used in bioremediation but unsuitable under high salt concentrations. In addition, these activities contribute significantly to metal contamination in natural and artificial water systems, including hypersaline environments (Tabak et al., 2005; Voica et al., 2016). Hypersaline habitats, often associated with high levels of different heavy metals due to their innate capability to concentrate ions and geological features, exemplified by brine‐rich areas of the Atacama Desert (among the world's primary lithium reserves) or other hypersaline environments like Searles and Mono Lakes in the Western USA with elevated arsenate levels, and several soda lakes in Central Asia showing high uranium concentrations (Voica et al., 2016). A coevolutionary relationship exists between haloarchaea and high concentrations of heavy metals, leading these microorganisms to develop various resistance mechanisms that allow them to thrive under metal stress (Bini, 2010; Kaur et al., 2006; Wang et al., 2004), with a broad range of tolerance varying by species (Leonard et al., 2001; Popescu & Dumitru, 2009; Sehgal & Gibbons, 1960). Generally, microorganisms employ five main mechanisms for heavy metal resistance: extracellular barriers, active transport of metal ions (efflux), extracellular sequestration, intracellular sequestration and reduction of metal ions (Bruins et al., 2000). Due to their industrial significance, this study chose nickel, chromium, lithium, cobalt and arsenic. Chromium, widely used in industrial processes like tannery and metallurgy, is recognized as a carcinogen for humans despite its favourable physical and chemical properties (Lukanova et al., 1996; Velusamy et al., 2020). Lithium has applications in the pharmaceutical sector (e.g., lithium carbonate for mood disorders like bipolar disorder) and battery technology (lithium‐ion batteries) (Boerlin et al., 2020; Mossali et al., 2020). Nickel is crucial in manufacturing rechargeable batteries for devices like cell phones (Majeau‐Bettez et al., 2011; Ovshinsky et al., 1993; Zhang et al., 2002) and electric vehicles. Cobalt finds applications in jewellery (Uter et al., 2014) and medical implants (e.g., joint replacements and coronary stents) and also serves in radiotherapy to produce gamma radiation for cancer treatment (Gyarmathy et al., 2008; Hansen et al., 2013). Arsenic, known for its toxicity and historically used in wood preservation, has limited industrial use and is declining in significance due to safer alternatives (Nelson, 1977). The oxidized species of these metals at high concentrations can induce severe cellular damage, disrupting cell membranes, altering enzymatic specificity, impeding cellular functions and damaging DNA (Bruins et al., 2000).

Based on experimental data, this work provides a comprehensive global perspective on the stress response of nine different halophilic archaea. That includes determining their tolerance range of optimal temperatures, salinities, oxidative stress and metal tolerance, focusing on the striking results obtained in the presence of lithium, to establish a rank of halophilic microorganisms with potential uses in bioremediation. Although more work is needed to elucidate the main molecular mechanisms involved in metal resistance in haloarchaea, these results represent an excellent starting point to develop environmentally friendly approaches to remove metals, particularly lithium, whose extensive use in batteries represents a real risk of environmental contamination.

## EXPERIMENTAL PROCEDURES

### Strains

The nine strains of haloarchaea selected were as follows: *Haloferax mediterranei* R4, *Haloferax gibbonsii* MA2.38 (Juez et al., 1986), *Haloferax volcanii* H1424 (Stroud et al., 2012), *Halorubrum californiense* AW8 (Sahli et al., 2020), *Halorubrum litoreum* (Cui et al., 2007), *Natrinema pellirubrum* AW13 (Sahli et al., 2020), *Natrinema altunense* F1 (Sahli et al., 2020), *Haloterrigena thermotolerans* BS7 (Sahli et al., 2020) and *Haloarcula sinaiiensis* (Javor et al., 1982).

### Culture media and stress conditions

The basal culture media employed for characterising halophiles under stress conditions consisted of 32.5% seawater (SW) containing 260 g NaCl, 32.5 g MgCl_2_ · 6H_2_O, 38 g MgSO_4_ · 7H_2_O, 7.6 g KCl, 0.54 g · CaCl_2_ · 2H_2_O per litre. This medium was supplemented with 5 g peptone and 1 g yeast as a source of nutrients. In all experiments, the turbidity of the media, reflecting cell density, was measured at a wavelength of 600 nm (OD_600_). Three biological replicates were used to ensure reproducibility, and the starting OD_600_ of all cultures was set to 0.02. Growth rates, doubling time and statistical analyses were calculated as described in Matarredona et al. (2021).

### Salinity and temperature stress

Initially, optimal values for salinity and temperature were determined separately. To assess the impact of salinity on growth, cells were cultivated in the basal medium with a range of seawater (SW) between 10 and 32.5% SW (w/v), with intervals of 10, 12.5, 15, 17.5, 20, 22.5, 25, 27.5, 30 and 32.5% by diluting of the initial recipe. The final concentration of each anion can be referenced in Table S1. Cells were grown at their optimal salinity over a range of 32–52°C (32, 37, 40, 42, 45 and 52°C) to investigate the effect of temperature on strain growth. A statistical descriptor, ‘tolerance coefficient’, was calculated by dividing the conditions where the culture maintained at least half its maximum growth by the conditions tested. This value aims to capture the breadth of the survival capability.

### Oxidative and heavy metal stress

Once salinity and optimal temperature were determined, oxidative stress was induced by adding H_2_O_2_ until the maximum concentration tolerated by the species was reached. The addition of hydrogen peroxide and heavy metals followed the procedure outlined in Matarredona et al. (2021). Cells were grown until reaching the mid‐exponential phase before adding the hydrogen peroxide.

In the analysis of metal resistance, five metals known for their toxicity to most living beings at high concentrations were tested: nickel (Ni^2+^), arsenic (As^5+^), cobalt (Co^2+^), chromium (Cr^6+^) and lithium (Li^+^). Metal salts used in the study included NiSO_4_, Na_2_HAsO_4_, CoCl_2_, K_2_CrO_4_ and LiCl. Stock solutions were prepared in distilled water and underwent sterilization through filtration with 0.22 μm pore size membrane filters. Different concentrations of metals were tested until the minimum inhibitory concentration (MIC) values were determined.

The growth reduction at 50% (GR_50_) rate was calculated for metal and oxidative stress conditions. That is defined as the concentration of metal or hydrogen peroxide at which the microorganism experiences a 50% reduction in its growth.

### Sample treatment for inductively coupled plasma mass spectrometry (ICP‐MS)

The six analysed strains that showed the highest tolerance to either lithium or chromium were selected for measuring the intracellular concentrations of main cations using ICP‐MS. The following procedure was carried out according to Matarredona et al. (2021). The halophilic archaea were independently cultivated in the previous culture medium at specific concentrations (Li^+^ 2 M: *Hfx. mediterranei*, *Hfx. volcanii*, *Hfx. gibbonsii*, *Nnm. altunense* and *Htg. thermotolerans;* Li^+^ 0.5 M: *Nnm. pellirubrum*). Initially, cells were grown until reaching the stationary phase. After pelleting the cells through centrifugation at 10,000×*g* for 10 min, they were washed multiple times with 20% sterile seawater. Half of the wet pellets underwent acidic mineralization to oxidise organic matter, solubilise metals and simplify the matrix. The samples were weighed, placed in acid‐washed 15 mL quartz digestion vials with Teflon‐lined caps and mixed with 4 mL of HNO_3_ and 1 mL of H_2_O. The mineralization was performed using the Ultrawave digestor from Milestone, following a specific microwave program: ramp to 100°C in 5 min, 15 min from 100 to 170°C, 10 min from 170 to 240°C and a 15‐min holding time at 240°C. Postmineralization, sample volumes were adjusted to 15 mL with Milli‐Q water. Each species' control culture (lithium‐free) was treated similarly and analysed to get a reference. The other half of the wet pellets were weighed, dried overnight at 80°C and re‐weighed to determine the dry biomass content. Concentration values obtained from ICP‐MS were extrapolated to mg/kg of dry biomass for each strain.

### Quantification of metal content by ICP‐MS

The elements were determined by ICP‐MS at the Research Technical Services of Alicante University. Calibration standards were prepared using different concentration solutions of PlasmaCal SCP33MS (SCP SCIENCE) in 1% nitric acid. An Agilent 8800 inductively coupled plasma mass spectrometer triple quadrupole (ICP‐MS QQQ), equipped with a micromist concentric nebulizer (Meinhard, Lyon, France), a Peltier cooled double‐pass spray chamber, standard torch and autosampler, was used for analysis. Data analysis was performed using a Mass Hunter workstation for ICP‐MS QQQ software (Agilent Technologies, Las Rozas, Madrid, Spain) to quantify the total concentration of elements in sample solutions. Two tune modes were sequentially employed to ensure proper ionization and interference removal, with an internal standard used for signal drift correction.

### Polar plot

The polar plot was rated on a scale of 1–10 points, encapsulating microorganism abilities to thrive within the proposed range for each of the evaluated scenarios. The score for each microorganism under each stressful environment was calculated using the following formula where *Vr* represents a specific value within a range (e.g., 42°C), *Gr* represents the growth rate observed at that particular value and *n* means the number of values in the range.

$$\sum_{0}^{n}\mathrm{Gr}\times \mathrm{Vr}.$$

Then, considering the highest score obtained among the nine species, the values were normalised to a 10‐point scale.

## RESULTS

### Salinity and temperature response of haloarchaea

**FIGURE 1:**
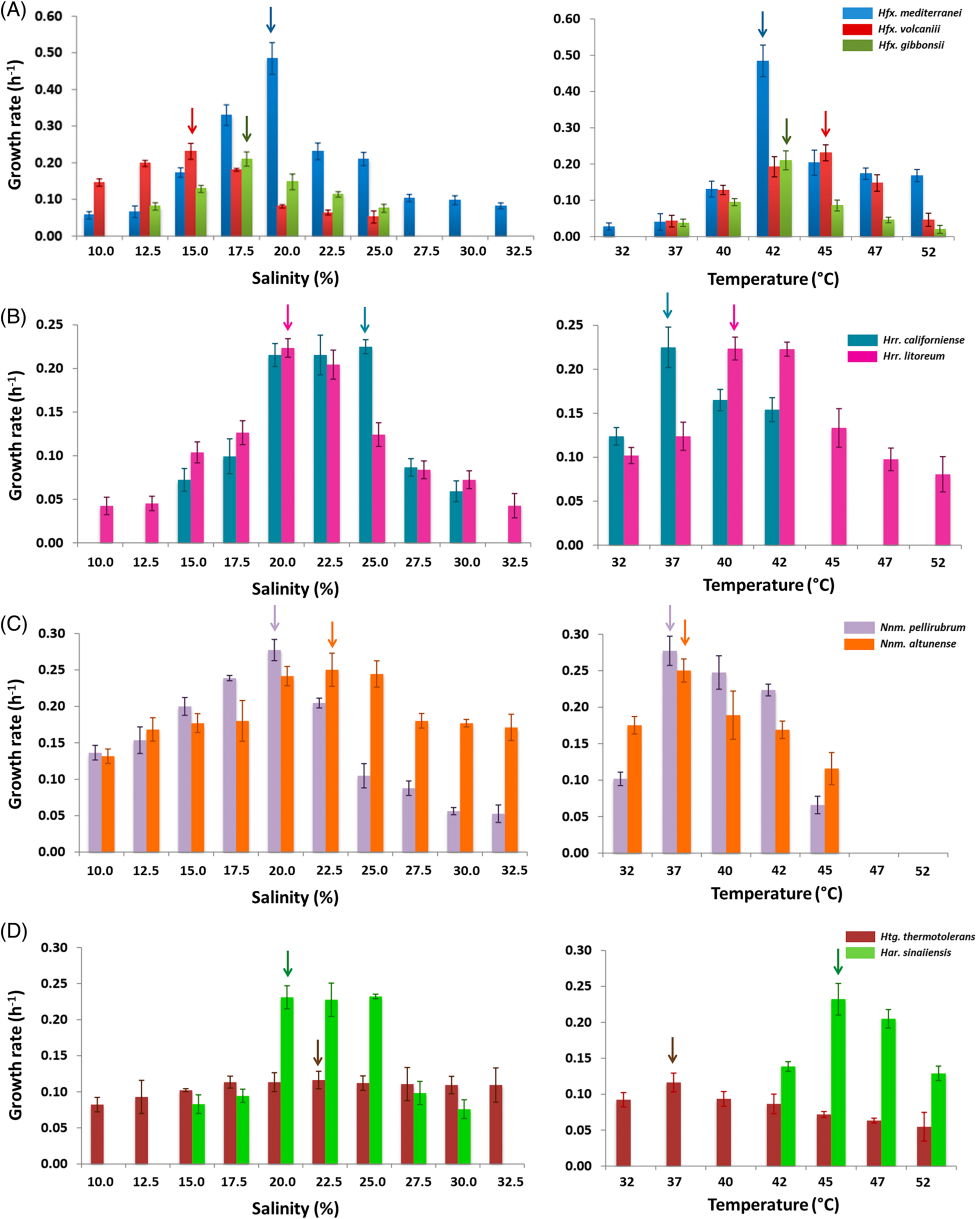
Effect of salinity and temperature on the growth rate of several haloarchaea. Values were obtained by varying a single parameter while keeping the remaining conditions at the determined optimum. (A) *Haloferax* genus; (B) *Halorubrum* genus, (C) *Natrinema* genus, (D) *Haloterrigena* and *Haloarcula* genus. The narrows reflect the maximal growth rate in each trial.

**FIGURE 2:**
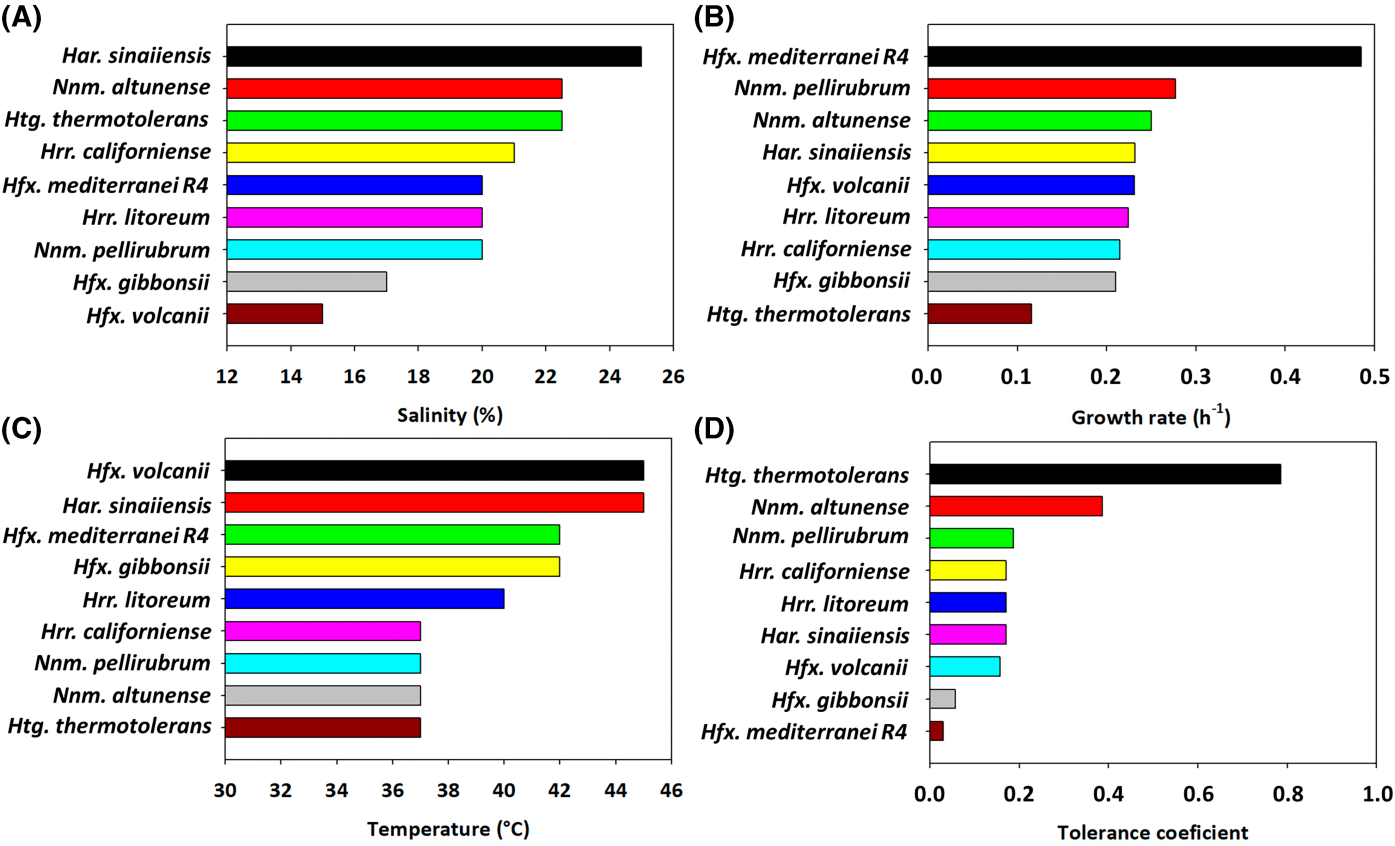
Classification of the nine haloarchaeal species evaluated based on the optimum exhibited for (A) salinity, (B) temperature, (C) growth rates and (D) tolerance coefficient.

**FIGURE 3:**
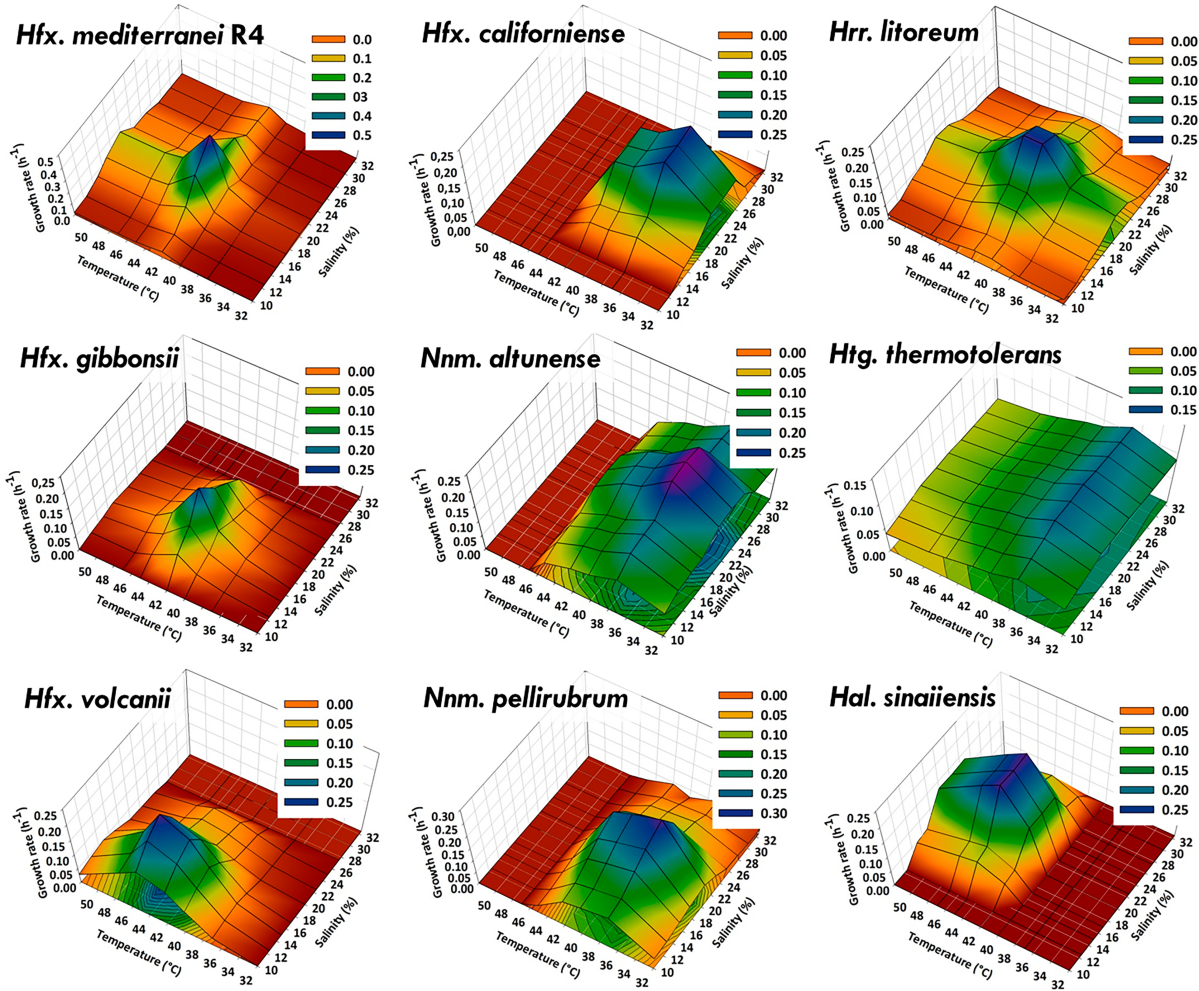
Topological plot representing the relationship between temperature, salinity and growth rate.

In their natural environment, haloarchaea typically survive in salinity ranges from 10% to 35% (w/v), with concentrations below 10% considered unviable for most species (Andrei et al., 2012; McGenity et al., 2000; Rodriguez‐Valera et al., 1983). Nevertheless, species within the same genus can display significant variations in their optimal salinity concentrations required for growth. Nine haloarchaeal species were tested across different salinity and temperature levels to assess their potential adaptability and identify optimal growth conditions. A standard growth curve was generated for each species under 70 different conditions (10 salinity levels × 7 temperatures), and growth descriptors were calculated (Figure 1). These results are discussed in more detail in Figures 2 and 3.

Within the nine haloarchaeal species analysed, *Haloferax* species, such as *Hfx. volcanii*, *Hfx. mediterranei* and *Hfx. gibbonsii*, emerged as robust candidates for studying environmental stress tolerance due to their well‐characterised biochemistry and molecular biology. Notable differences were observed among the species: *Hfx. volcanii* exhibited good growth even at 10%, with the lowest optimal salinity (15%) among all species but required a slightly higher optimal temperature than *Hfx. gibbonsii* and *Hfx. mediterranei*. This behaviour perfectly aligns with the information provided in other works (Bidle, 2003; Mullakhanbhai & Larsen, 1975; Tittes et al., 2021; Torreblanca et al., 1986). *Hfx. gibbonsii* showed a narrow salt tolerance, with growth only between 12.5% and 25%. To date, these are the only studies conducted to determine optimum salinity and calculate growth kinetic parameters with this species. By contrast, *Hfx. mediterranei* demonstrated high adaptability, growing in all 70 conditions, demonstrating high adaptability to the environment. However, its growth remained near the maximum only in a narrow range of conditions, sharply decreasing outside the optimal area. That is evident in Figure 3A, where a three‐dimensional view of the relationship between temperature, salinity and growth is presented.

The genus *Halorubrum*, widely distributed in hypersaline environments, was represented in this study by *Hrr. californiense* and *Hrr. litoreum*, which thrived at salinity levels between 20% and 25%, and temperatures of 37–40°C. This positions *Halorubrum* slightly above *Haloferax* in salinity requirements but lower in thermophily (Figure 2A,B). Both species showed similar growth rates to other haloarchaea, with a doubling time of around 3 hours, consistent with previous findings (Robinson et al., 2005). *Hrr. litoreum* demonstrated higher tolerance to temperature and salinity compared with *Hrr. californiense*. It was one of the only three species that grew under all the tested conditions. However, similar to *Hfx. mediterranei*, the growth rate was dramatically affected when the optimal ranges for these parameters were exceeded (Figure 1B). These results align with earlier studies, which report *Hrr. californiense* thriving in salinities from 14.61% to 29.2% and temperatures between 25 and 42°C, with an optimum at 37°C (Pesenti et al., 2008). Conversely, similar patterns were observed in *Halorubrum salinarum* and *Halorubrum saccharovorum*, with optimal growth at salinities of 20%–25% and temperature up to 55°C (Cui et al., 2007; Pesenti et al., 2008; Robinson et al., 2005).

The genus *Natrinema*, common in hypersaline environments, was represented by two strains, *Nnm. pellirubrum* and *Nnm. altunense;* both chemo‐organotrophs are found in alkaline high salt environments (Gupta et al., 2015). Like *Halorubrum*, these species displayed optimal growth at salinities between 20% and 22.5%, though *Nnm. altunense* exhibited exceptional growth across all tested salinity levels, maintaining growth rates close to the optimum (Figure 1C). These values align with an earlier essay in which *Nnm. altunense* strain AJ2T was reported as an extremely halophilic archaeon growing at 10%–25% NaCl aerobically cultivated at 37°C (Xue‐Wei et al., 2005). Both species encountered difficulties growing above 42°C, with an optimal temperature around 37°C, making them the species with the lowest temperature requirements in this study and classifying them as mesophiles. That contrasts with previous reports where *Nnm. pellirubrum* grew to 57°C, with an optimum at 51°C and a doubling time of 1.87 h (Robinson et al., 2005). However, there seems to be a general trend among *Natrinema* species to prefer 37°C as the optimal temperature. The doubling times at their optimal points were 2.5 h for *Nnm. pellirubrum* and 2.77 h for *Nnm. altunense* (Table S2). Comparisons with other *Natrinema* strains, such as *Natrinema ejinorense*, *Natrinema versiforme*, *Natrinema halophilum*, *Natrinema salinisoli* and *Natrinema amylolyticum*, revealed a typical pattern: growth at low NaCl concentrations (around 10%), with an optimum range near 20%, and a standard preference temperature close to 37°C, which is considerably lower than other haloarchaea species studied in this research (Bao et al., 2022; Castillo et al., 2006).

Finally, two independent species were utilised in this study. Firstly, it was examined *Har. sinaiiensis*, a Haloarculaceae family member isolated from Red Sea Sabkha Gavish brine samples (Javor et al., 1982). This microorganism displayed notable specificity, thriving in a limited range of conditions characterised by salinities between 15% and 30% and temperatures between 42 and 52°C, with optimum in 25% and 45°C. No growth was observed outside of this specified area. These findings reveal strong halophilic and thermophilic preferences (Figure 2A,B). These ranges closely resemble those of *Haloarcula marismortui*, *Haloarcula japonica* and *Haloarcula vallismortis* (Robinson et al., 2005; Takashina et al., 1990; Thombre et al., 2016). Despite this specificity, *Har. sinaiiensis* displayed a doubling time of around 3 h under optimal conditions, much faster than *Har. marismortui*, which achieved doubling times of nearly 24 h (Thombre et al., 2016).

The behaviour of *Htg. thermotolerans* was explored as the sole representative of the diverse genus *Haloterrigena*, which belongs to the Natrialbaceae family. This species, known for being chemoorganotrophic, aerobic and halophilic, requiring at least 2 M NaCl (Takashina et al., 1990), demonstrated extreme adaptability in our study (Figure 2D). Surprisingly, it grew in all tested scenarios with nearly the same growth rate, regardless of salt concentrations or temperature values. Only slight optima were detected at a salinity of 22.5% and a temperature of 37°C. Along with *Hfx. mediterranei* and *Hrr. litoreum*, *Htg. thermotolerans* was one of the few species that could grow in the 70 conditions (Figure 2A–D). Despite its high tolerance, it had the longest doubling time, around 6 h (Table S2). This aligns with previous studies, which reported its tolerance to temperatures up to 60°C, with an optimal growth around 50°C (Montalvo‐Rodríguez et al., 2000). Additionally, other studies indicated 33.5% salinity and 37°C as ideal conditions for producing C50 carotenoids in this species (Kesbiç & Gültepe, 2022).

It should be noted that this is the first work where growth kinetic parameters have been calculated for nine different haloarchaea in a total of 70 conditions, combining different temperatures and salinities since most of the previous studies are based on the species descriptions. All these data presented above separately for each species are globally compared in Figures 2 and 3. Figure 2 summarises the key findings, ranking the nine studied species based on the optimal salinity, temperature, growth rate and tolerance. *Hfx. volcanii* had the lowest optimal salinity (15%), while *Har. sinaiiensis* had the highest (25%) (Figure 2A). In terms of temperature, *Hfx. volcanii* and *Har. sinaiiensis* were the most thermophilic species (45°C), while four species showed optimal growth at 37°C (Figure 2B). *Hfx. mediterranei* exhibited the highest growth rate value (0.485 h^−1^), making it promising for biotechnological applications, while *Htg. thermotolerans* had the lowest (0.116 h^−1^) (Figure 2C). However, species with higher growth rates, like *Hfx. mediterranei*, experienced a sharp decline outside optimal conditions, affecting their adaptability. By contrast, *Htg. thermotolerans* and *Hrr. altunense* showed steady growth across a wider range (Figure 2D).

Figure 3 further highlights this adaptability, showing an inverse correlation between growth rates and tolerance coefficients (data not shown). In this context, microorganisms with higher growth rates in well‐defined areas of temperature and salinity tend to experience a sharp decline in growth outside of those optimal conditions, resulting in a loss of adaptability. On the contrary, species with lower growth rates, such as *Htg. thermotolerans*, maintained growth in 80% of the tested conditions, outperforming *Hfx. mediterranei* in 56 out of 70 scenarios, demonstrating superior adaptability across a range of temperatures and salinities.

### Oxidative stress in haloarchaeal strains

**FIGURE 4:**
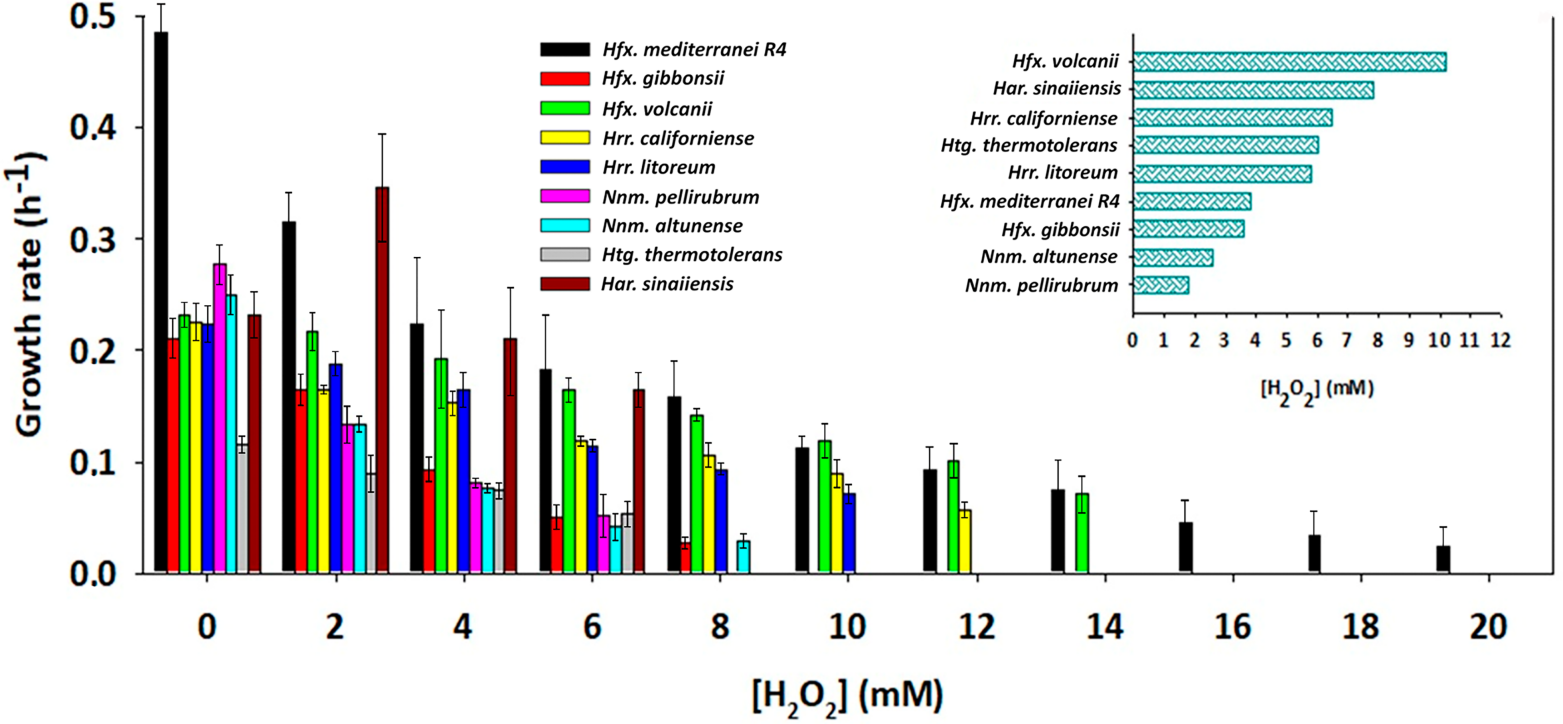
Growth rates of haloarchaea under different hydrogen peroxide concentrations to induce oxidative stress. The upper right corner displays a ranking indicating the GR_50_ value for each species under H_2_O_2_ treatment. GR_50_ is the concentration required to reduce the growth rate below half that of the control.

Haloarchaea have evolved protective mechanisms to mitigate the detrimental effects of oxidative stress caused by extreme environmental conditions such as desiccation, radiation and high salinity. These microorganisms produce antioxidant enzymes, like superoxide dismutase and catalase, and synthesise carotenoid pigments to neutralize reactive oxygen species (ROS) (Jantzer et al., 2011). They also possess efficient DNA repair mechanisms to protect their genetic material, which makes them valuable for biotechnological applications like bioremediation in saline and polluted environments (Dutta & Bandopadhyay, 2022; Haque et al., 2020; Mainka et al., 2021). Despite the limited research on the oxidative stress tolerance of the nine selected haloarchaea, this study provides insights into how they withstand peroxide radicals by exposing them to hydrogen peroxide during mid‐exponential growth. As shown in Figure 4, the effect of varying hydrogen peroxide concentrations on the growth rates of these strains reveals interesting patterns. The upper right corner of this plot also presents a ranking that includes the GR_50_ value for each species, representing the stressor concentration required to reduce the growth rate to half of the control. First, it should be highlighted that *Hfx. mediterranei* is the only species that exhibits significant growth across all concentrations, even thriving (0.024 h^−1^) at levels as high as 20 mM. This level of tolerance is close to 25 mM of *Halobacterium salinarum* NRC1 was obtained in a complex medium (Kaur et al., 2010; Matarredona et al., 2020) and surpassed the 16 mM observed in previous studies, using the same strain (Matarredona et al., 2021) in a defined medium with ammonium and glucose as main macronutrients.

Interestingly, it appears that *Hfx. mediterranei* also shows greater resistance in the complex medium than in the defined medium, underscoring the significant influence of the growth environment and the availability of nutrients. This finding underscores the importance of conducting assays in a defined medium resembling natural conditions rather than nutrient‐rich media. Despite this high tolerance, it is essential to emphasise that *Hfx. mediterranei* experiences a significant reduction in its maximum growth when exposed to low concentrations of H_2_O_2_, losing more than 50% of its growth at only 4 mM. This observation aligns closely with the trends observed in this species' salt/temperature section. On the contrary, *Hfx. volcanii*, despite having a low basal growth rate, manages to sustain it above 50% even at values as high as 8 mM of hydrogen peroxide. Furthermore, it grew slightly faster than *Hfx. mediterranei* at concentrations of 10–12 mM. Shifting the focus to the behaviour of the other species, the *Halorubrum* genus also displayed an interesting performance, with 10 mM and 12 mM H_2_O_2_ as minimum inhibitory concentration (MIC) for *Hrr. litoreum and Hrr. californiense*, respectively. The growth rate of these microorganisms also remained above 50% until reaching the MIC. Also interesting was the behaviour of *Har. sinaiiensis*, which displayed a better growth rate at 2 mM of the stressor than the control, and then sharply dropped from almost the maximum to nullity in the tight change between 6 and 8 mM. On the other side, both representatives from the *Natrinema* genus exhibited the highest sensibility to oxidative stress, ceasing to grow completely between 2 and 3 mM.

### Metal stress response of haloarchaea

**FIGURE 5:**
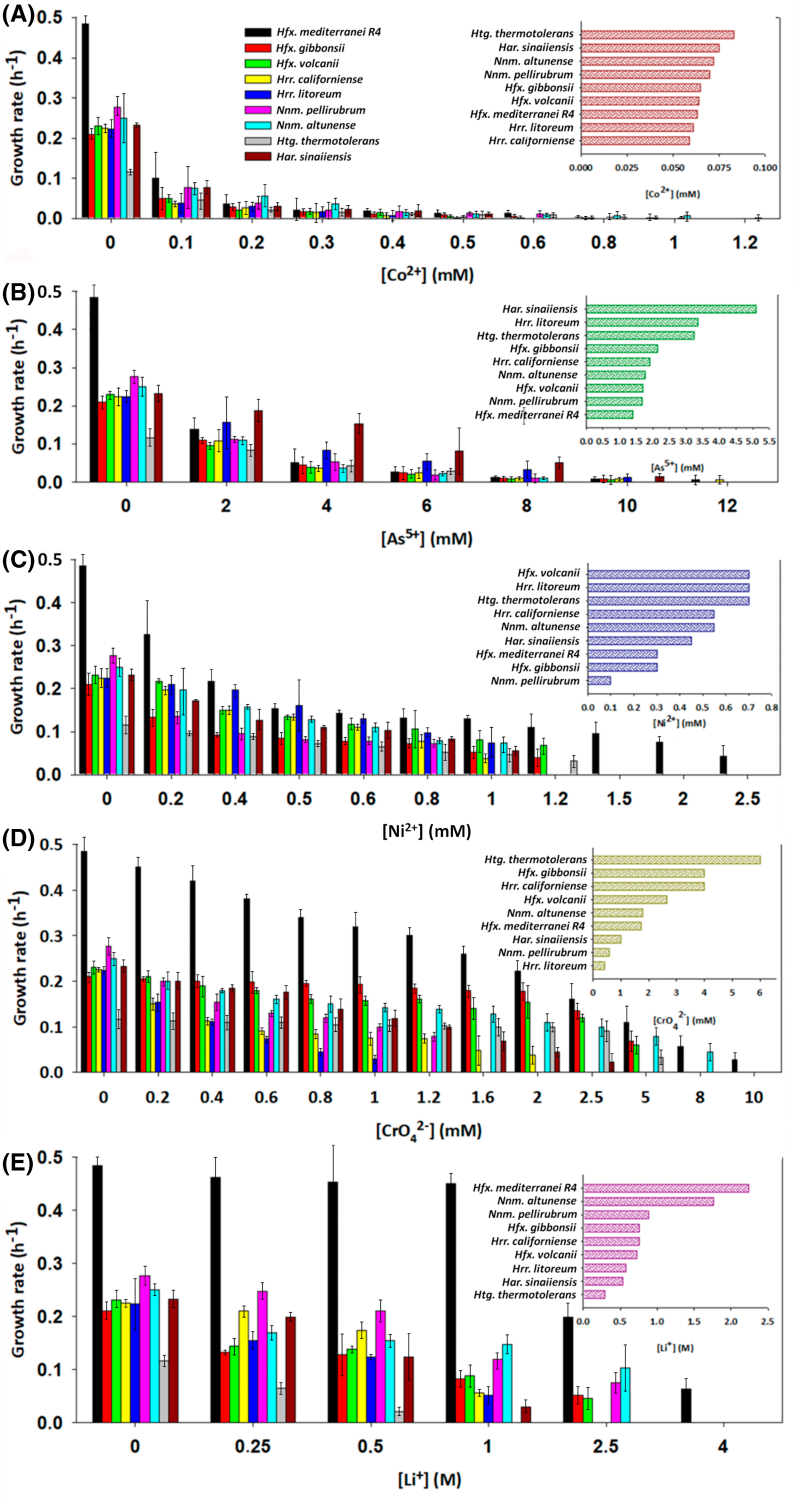
Growth rates of haloarchaea under different metal concentrations to induce stress. (A) cobalt stress; (B) arsenic stress; (C) nickel stress; (D) chromium stress; (E) lithium stress. The upper right corner displays a classification of the nine analysed species in function of their GR_50_.

Heavy metal pollution represents a significant environmental challenge due to the toxic effects associated with these elements. As previously highlighted, hypersaline environments have intricate connections with various heavy metals due to geological factors or human activities. Within these environments, halophilic microorganisms have developed different strategies to thrive in the presence of metals (Voica et al., 2016) and the response to toxic metals varies depending on the specific characteristics of the biotope to which they have adapted. That makes hypersaline environments a natural scenario for studying the response of halophilic archaea to metal exposure. In this study, the response of these haloarchaea to heavy metals was assessed by determining the MIC and GR_50_ for five specific heavy metals: nickel (Ni^2+^), arsenic (As^5+^), cobalt (Co^2+^), chromium (Cr^6+^) and lithium (Li^+^) (Table 1 and Figure 5). The observed behaviour of these haloarchaea exhibited variations when exposed to different heavy metals. From a general point of view, all nine strains demonstrated a degree of tolerance to Ni^2+^, As^5+^, Co^2+^, Cr^6+^ and Li^+^. The subsequent results will delve into the specific physiological responses of individual species to these heavy metal stressors.

**TABLE 1:** MICs of five metal ions tested against the nine species of haloarchaea.

Microorganism	MICs (mM)				
	As^5+^	Cr^6+^	Co^2+^	Ni^2+^	Li^+^
*Hfx. mediterranei* R4	12	10	1	2.5	4000*
*Hfx. gibbonsii*	10	5	1	1.2	2500**
*Hfx. volcanii*	10	5	1	1.2	2500**
*Hrr. californiense*	12	2	0.5	1.0	1000
*Hrr. litoreum*	10	1	0.5	1.0	1000
*Nnm. pellirubrum*	8	1.2	1.0	0.8	2500**
*Nnm. altunense*	8	8	1.2	1.0	2500**
*Htg. thermotolerans*	6	5	0.8	1.2	500
*Har. sinaiiensis*	10	2.5	0.5	1	1000

*Note*: Three different biological replicates were performed to determine the MICs.

* In this culture medium, NaCl (4 M) is replaced by LiCl.

** The culture medium contained 2 M LiCl and 2 M NaCl.

#### Cobalt

Across all nine species, cobalt emerged as the heavy metal to which they exhibited the highest sensitivity, suggesting its significant toxicity to these haloarchaea. Cobalt is generally essential for microorganisms as it is a component of coenzymes and plays a crucial role in specific metabolic reactions. However, at elevated concentrations, cobalt's negative impact became evident, with most species unable to tolerate levels exceeding 1 mM. The results revealed minimal variations between species, as all displayed potent inhibition at the lowest tested concentration, resulting in very similar GR_50_ values of approximately 0.1 mM (Figure 5A). Co^2+^ is the metal to which all species have shown the most significant difficulties in starting to grow, delaying or inhibiting the onset of the exponential phase. The MIC values detailed in Table 1, consistently close to 1 mM, align with previous findings indicating that this concentration is the threshold preventing growth in many haloarchaea (Nieto et al., 1987). Given the presence of cobalt in industrial wastewater, its removal is essential due to its serious health effects and environmental hazards. In terms of future applications, a key biotechnological opportunity lies in exploring the biosorption capability of haloarchaea for remediating Co^2+^ contaminated waters in natural environments. Besides being relevant in bioremediation, it is also useful in the mining industry to produce cobalt (Matarredona et al., 2021; Schippers et al., 2014). Among the species analysed, the genera *Haloferax* or *Natrinema*, especially *Nnm. altunense*, could be considered for further studies because they showed the highest capability to hold some growth at high concentrations of cobalt values, typically around 1 mM.

#### Arsenic

Arsenic contamination is a significant environmental concern due to its toxicity and widespread presence in natural water sources, even at very low concentrations. Arsenic can exist in various oxidation states, with arsenate (As (V)) and arsenite (As (III)) being the most common forms in natural waters. Inorganic arsenic, especially trivalent arsenic, is generally toxic to microorganisms, affecting cell viability and growth. In this study, most haloarchaea exhibited a MIC around 10 mM, including species like *Hfx. gibbonsii*, *Hfx. volcanii* and *Hrr. litoreum*. The highest values were observed for *Hfx. mediterranei* and *Hrr. californiense*, with 12 mM, while the lowest value was recorded for *Htg. thermotolerans* (Table 1). Remarkably, *Har. sinaiiensis* displayed an unexpected response, achieving the best GR_50_ value and maintaining half its potential growth up to 5 mM. Despite having a relatively low growth rate in the absence of the stressor, it exhibited the highest growth rate values at concentrations between 2 and 8 mM of arsenic, outperforming other species that already displayed limited growth at these concentrations (Figure 5B). When considering bioremediation candidates, it is crucial to focus on the MIC value and the growth rate profile concerning the concentration of the toxic agent. In this context, although *Har. sinaiiensis* may not survive at concentrations as high as other organisms, but it maintains excellent performance at intermediate to high arsenic concentrations. Therefore, it is proposed as a promising bioremediation agent for contaminated saline waters with this metal. On the contrary, previous work showed that 20 mM is the lowest metal concentration preventing the growth of *Hbt. salinarum* and *Hfx. volcanii* (Gelsinger & DiRuggiero, 2018).

#### Nickel

In previous studies, it was observed that members of the genus *Haloferax*, such as *Hfx. mediterranei*, *Hfx. volcanii* or *Hfx. gibbonsii* can tolerate similar concentrations of nickel, around 2.5 mM. However, this tolerance is not a common trait among all haloarchaea. For instance, *Haloarcula* sp. can only grow in 0.1 mM Ni^2+^, while *Har. sinaiiensis* can tolerate up to 1 mM (Pei et al., 2020). In the current study, both MIC and GR_50_ values revealed that all species exhibited a similar response to nickel, with MIC values around 1 mM (Table 1 and Figure 5C). In partial agreement with the analysed literature, *Haloferax* exhibited slightly less sensitivity than the other genus, showing some growth even at 1.2 mM. Within the *Haloferax* genus, only *Hfx. mediterranei* was able to grow at the referenced 2.5 mM. It is worth noting that despite not surviving at concentrations higher than 1.2 mM, *Hfx. volcanii* maintained excellent growth at concentrations around 1 mM compared with the control, ranking first in GR_50_. However, under any conditions, its growth exceeds that of *Hfx. mediterranei*. Therefore, *Hfx. mediterranei* is considered the most suitable biotechnological candidate for treating saline and brackish waters contaminated with nickel. That is particularly relevant in the industrial sector, especially considering the growing importance of nickel‐hydrogen batteries for large‐scale energy storage (Chen et al., 2018). On the opposite side, the microorganism that showed the highest sensitivity to Ni^2+^ was *Nnm. pellirubrum*; it could not survive at concentrations higher than 0.8 mM.

#### Chromium

Chromium is one of the primary environmental pollutants from various anthropogenic activities, notably in stainless steel manufacturing and leather tanning (Cheng et al., 2023; Pei et al., 2020). Given its widespread applications, chromium has become a significant concern due to its pervasive presence in various sectors. Therefore, it is crucial to investigate the response of haloarchaea to chromium and determine their maximum tolerance levels. In this study, nine haloarchaea were exposed to concentrations of up to 10 mM of CrO_4_^2−^ (Cr (VI)). Unlike other metals such as nickel or cobalt, where all species behaved similarly, the response to chromium varied significantly, with MICs differing by up to an order of magnitude. From the results, *Hfx. mediterranei* emerges as the microorganism that supports the highest concentrations of chromium, reaching up to 10 mM (Table 1). Then, closely is *Nnm. altunense*, displaying notable stress tolerance at 8 mM. The MIC values of the *Natrinema* species differed significantly, from those of *Hrr. litoreum* is the most sensitive microorganism to chromium, tolerating only 1 mM. In terms of growth, *Nnm. altunense* showed remarkable adaptability, maintaining a growth rate close to the control at almost all the tested concentrations and ranking in the first place of GR_50_ values. However, despite this resilience, it could not exhibit a growth more significant than that of *Hfx. mediterranei* in any of the conditions assayed. These findings match with those reported by Nieto et al. (1987).

#### Lithium

Lithium, a malleable metal which plays a key role in various industrial applications, is particularly valuable in energy management and is highly prevalent in most hypersaline environments. In this study, all tested haloarchaea could grow in the presence of this metal at concentrations several orders of magnitude higher than other metals. Remarkably, *Hfx. mediterranei* displayed an extraordinary ability to grow in a culture medium without NaCl, entirely replaced by LiCl; it could thrive even at 4 M of LiCl (Figure 5E). It is worth noting that lithium did not appear to be a significant stressor for this species, as it could adapt to living without NaCl. Furthermore, it is noteworthy that at 4 M of LiCl, the microorganism took approximately 2 weeks to initiate growth.

Focusing on the case of lithium, we see that not only all species have a higher tolerance value but also *Hfx. mediterranei*, the most tolerant, can grow at reasonable growth rate at 2.0 M LiCl. It is important to note that cultures with 2.0 M LiCl had replaced 2.0 M NaCl with LiCl, meaning the medium contained 2 M LiCl and 2 M NaCl.

The results obtained in this part of the findings are extremely valuable for promoting the biotechnological applications of haloarchaea. There are no precedent works where such a broad range of haloarchaea has been subjected to five different contaminants at such broad concentration ranges in a single study, providing unprecedented insights. From the data obtained, the evaluation of each organism's suitability in response to various metals has been assessed by compiling two characteristics: (1) the ability to exhibit some growth at the highest possible concentrations of the different stressors and (2) the ability to maintain growth as close as possible to the control throughout the entire tolerance range. In a broader context, the metals under scrutiny can be categorised in terms of toxicity over the analysed strains, with cobalt, nickel, arsenic, chromium and lithium following a gradient of decreasing toxicity. Considering the potential application of each species as a bioremediation agent, they should not only thrive in highly polluted environments but also possess the capability to interact with the toxin, leading to its transformation, blockade and removal from the system. In this regard, a final research section was proposed to assess the interaction between six of the nine haloarchaea species and lithium to determine their ability to thrive in high lithium concentrations, metabolize it and incorporate it into biomass.

### Analysis of the tolerance of haloarchaea by ICP‐MS

**FIGURE 6:**
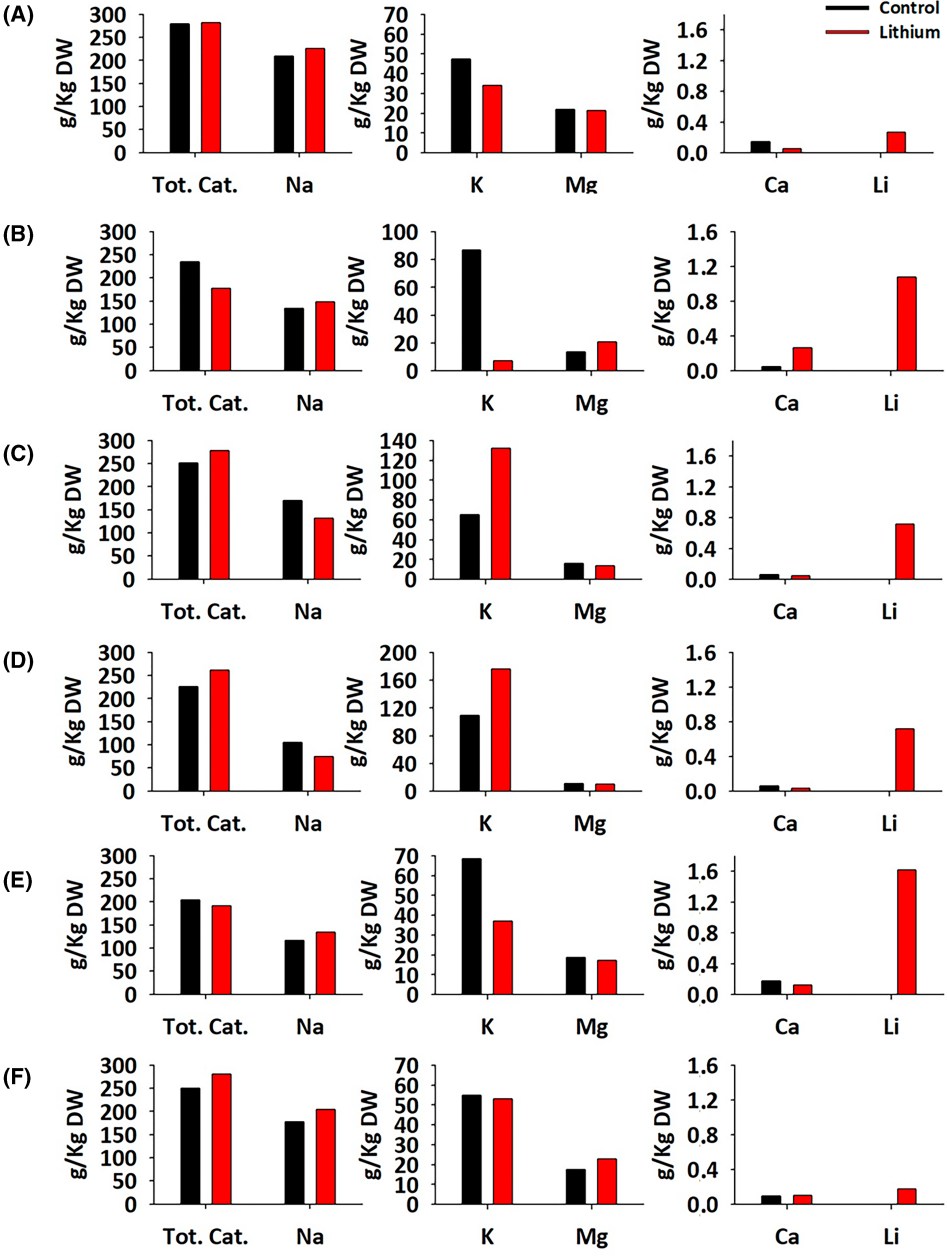
Variations in the five significant cations in the biomass of the six haloarchaea were determined by ICP‐MS. Black bars represent the control cultures, and red bars represent the cultures grown in lithium. (A) *Hfx. mediterranei*, (B) *Hfx. volcanii*, (C) *Hfx. gibbonsii*, (D) *Nnm. altunense*, (E) *Nnm. pellirubrum*, (F) *Htg. thermotolerans*. DW—dry weight.

ICP‐MS was employed to quantitatively analyse the presence of five significant cations (sodium, potassium, magnesium, calcium and lithium) in six selected haloarchaea out of the nine species studied (Table S3). The chosen species, included *Hfx. mediterranei*, *Hfx. volcanii*, *Hfx. gibbonsii*, *Nnm. altunense*, *Nnm. pellirubrum* and *Htg. thermotolerans*, were selected according to their growth rate and tolerance, as previously described. Each species was subjected to two conditions and their experimental replicates: the control, consisting of cells without any added metal, and the sample with the corresponding quantity of lithium for each species. The specific lithium concentrations added are detailed in the Experimental Procedures section. A summarized overview of the main results is displayed in Figure 6.

This section yields crucial insights, delineating three distinct scenarios based on the behaviour of different species:

1. *Hfx. mediterranei*: This species demonstrates an exceptional ability to thrive in environments with extreme lithium concentration; however, it contains surprisingly low lithium levels in its biomass (0.266 g/kg), ranking as the second lowest among the six species analysed. This observation aligns with known molecular mechanisms for toxic resistance, emphasising the role of three mechanisms preventing toxins from penetrating the cell interior (Cheng et al., 2017). Some studies have explored the S‐layer and exopolysaccharides (EPS) of haloarchaea, which vary in thickness and composition among species. Archaeal S‐layers can reach up to 70 nm and typically have a hexagonal lattice structure with a spacing distance of 12–16 nm (Ellen et al., 2010; Rodrigues‐Oliveira et al., 2017; Sleytr et al., 2014). Specific structural characteristics also vary, such as *Hbt. salinarum* and *Hfx. volcanii* have lattice constants around 15 and 16.8 nm, respectively, while *Hfx. mediterranei* presents a distinctive, thicker glycoprotein layer (Esclapez et al., 2013; Kessel et al., 1988; König et al., 2004; Trachtenberg et al., 2000). Therefore, the capability of *Hfx. mediterranei* to survive in high lithium concentrations may be due to the composition and greater thickness of its S‐layer. Additionally, secondary data extracted from the current study support this hypothesis. During dry weight (DW) determinations of the six species analysed, it was observed that all the wet biomasses had a similar dry residue content of approximately 36% (w/w). However, the wet biomass of *Hfx. mediterranei* consistently yielded values close to 27%, indicating its superior capability to retain water around the cell. Therefore, it can be assumed that its S‐layer and EPS envelope are more spread out in the space surrounding the cell than the other species. This feature could also explain the general high tolerance against most of the stress sources that haloarchaea displays.
2. *Hfx. volcanii* and *Nnm. pellirubrum*: These species showed a higher competence to accumulate substantial amounts of lithium (up to 1.0 and 1.6 g/L, respectively). However, they exhibit relatively high GR_50_ values, around 1 M. This suggests that, even when a portion of lithium breaches the S‐layer and enters the cell, the cellular machinery of these organisms can still carry out their routines with relative success even in the presence of this cation.
3. *Htg. thermotolerans* exemplifies a third possible situation. This species appears to be much more sensitive to small concentrations of lithium than the others, as it shows the lowest GR_50_ value and cannot thrive in concentrations exceeding 0.5 M. It can be assumed that at the molecular level, neither its cell envelope can prevent lithium from entering the cell nor can its cellular machinery tolerate the presence of even small concentrations of this element, leading to the cessation of growth even when containing very little lithium inside.

Besides the valuable information regarding lithium content obtained from the ICP analysis, the analysis of the remaining cations reveals fascinating insights into the effect of this metal at the cellular level. When assessing the total cation content, most species generally have no significant differences between the control and condition. Only in the cases of high lithium accumulation mentioned for *Hfx. volcanii* and *Nnm. pellirubrum*, a slight decrease in positive charges can be seen compared with the control. However, when analysing the potassium content, a substantial decrease is noticeable in these two species, particularly in *Hfx. volcanii*. This decline justifies the observed slight reduction in total cations. This adjustment in potassium levels is a compensatory mechanism to counterbalance the increased positive charges inside the cell, especially notable in *Hfx. volcanii*, as a reminder, displayed the lowest halophilicity among the nine species. The rest of the analysed cations remained similar between controls and conditions, except for an unusually high calcium content in *Hfx. volcanii* grown in lithium, possibly attributable to osmotic disturbances induced by the elevated presence of the metal.

## DISCUSSION

This study highlights the resilience and adaptability of halophiles, specifically haloarchaea, which make them promising candidates for biotechnological applications, particularly in bioremediation. The response of nine different haloarchaea species to variations in temperature, salinity, hydrogen peroxide and the presence of five industrially significant heavy metals was investigated, providing valuable experimental data on their potential biotechnological applications.

The results of this work showed that *Hfx. mediterranei*, *Hfx. volcanii* and *Hfx. gibbonsii* exhibited a high degree of selectivity in terms of optimal growth conditions. These species maintained near‐maximal growth rates in a narrow range of salinity and temperature, with minor fluctuations significantly reducing their growth rates rather than completely inhibiting growth. In this sense, these organisms exhibit two distinct metabolic models: one enabling rapid thriving when conditions fall within a narrow optimal range and another addressed to survival when environmental conditions deviate from these limits. Conversely, *Htg. thermotolerans* and *Nnm. altunense* demonstrated nearly constant growth rates in all assays, indicating a more generalist behaviour. A discernible trend emerges from this observation, suggesting high maximal growth rates, as observed in *Hfx. mediterranei* are achievable only within a narrow range of conditions, whereas microorganisms with a slower metabolism, like *Htg. thermotolerans*, can maintain their maximal growth rates across broader ranges.

Among the species tested, *Hfx. mediterranei* exhibited the highest growth rate under optimal conditions, reaching 1.43 duplications per hour—nearly double that of the following fastest‐growing species (Figure 2C). This fast growth rate and its ability to adapt to various environmental conditions underscore why *Hfx. mediterranei* is widely studied as a model extremophile. The higher growth rates obtained in this study, compared with previous research (1.43 vs. 2.15 h) (Matarredona et al., 2021), may be attributed to using a complex medium, which appears to enhance its performance relative to defined media. However, despite its high growth rate, *Hfx. mediterranei* ranked only fifth in terms of tolerance, according to Figure 2D. This result highlights a key finding: although *Hfx. mediterranei* thrives across diverse extreme conditions; its growth is significantly reduced outside its optimal range. That suggests that while this species can survive extreme environments, it does so less efficiently than other haloarchaea that maintain more consistent performance across broader environmental ranges.

In general, the findings of this study reveal the potential tolerance and the gradual loss of growth for the nine selected species in the presence of a strong oxidative stress source like H_2_O_2_. *Hfx. mediterranei* stood out as highly resilient under hydrogen peroxide exposure, maintaining significant growth even at 20 mM H₂O₂, making it the most tolerant species tested. For bioremediation strategies, two key criteria are considered: achieving the highest possible growth rate without stress and maintaining that rate across a broad range of conditions. Based on these criteria, both *Hfx. mediterranei* and *Hfx. volcanii* are proposed as excellent candidates for treating wastewater or contaminated soils with high levels of oxygen radicals.

Equally impressive is the ability of several species (*Hfx. volcanii*, *Hfx. gibbonsii*, *Nnm. pellirubrum*, *Nnm. altunense*) to grow by gradually replacing NaCl with LiCl. This behaviour in *Hfx. mediterranei* has been observed before, showing unexpected results. Surprisingly, this microorganism demonstrated superior growth rates to the control at low lithium concentrations, even thriving at up to 0.5 M LiCl (Matarredona et al., 2021). It is crucial to note that this study and current research represent the only available data on haloarchaea's growth analysis in the presence of lithium, one of the most important elements in the 21st century. Although there are some studies performed with halotolerant Bacteria, such as *Micrococcus luteus* or *Bacillus pumilus*, their lithium tolerance only reaches 30 g/L (0.7 M approximately), concentration 5‐fold lower than the lithium tolerated by different haloarchaea (Günan‐Yücel et al., 2021; Martínez et al., 2021). It appears that the unique composition and thickness of their cell envelopes play a crucial role in each species' extraordinary resistance to heavy metals. However, it is not yet possible to conclude if this ability to grow at high lithium concentration is an intrinsic characteristic only in haloarchaea or archaea in general. In any case, these results are astonishing. Despite the increasing use of microorganisms in metal recovery, information regarding lithium recovery still needs to be improved (Tsuruta, 2005). In recent years, the demand for lithium has increased exponentially due to its applications, such as in rechargeable batteries for various portable electronic devices (Owen, 1997; Wakihara, 2001). Based on all these promising results, especially with the haloarchaeon *Hfx. mediterranei*, proteomic approaches, among others, would help to understand the mechanisms involved in tolerance and thus elucidate the strategy followed by this haloarchaeon to adapt to Li^+^ and even grow without NaCl when LiCl replaces the NaCl in the culture medium.

The results from ICP‐MS demonstrated that tolerance and bioremediation capability are not synonymous. While *Hfx. mediterranei* exhibited the highest tolerance over lithium, it could not act as a bioremediation agent since it rejected the toxic substance and was not able to accumulate and lock it. Conversely, *Nnm. pellirubrun* was not exceptionally tolerant with lithium, but its envelope was highly permeable to the cation, accumulating up to 1.6 g/kg, while its cellular machinery continued functioning even at very high concentrations of the toxic substance. Results obtained through ICP‐MS add an additional layer of understanding by revealing that tolerance does not always translate into adequate bioremediation capacity. Further in‐depth studies are needed to elucidate in more detail the molecular mechanisms of adaptation of these haloarchaea to stress conditions to exploit their potential applications, especially in bioremediation.

**FIGURE 7:**
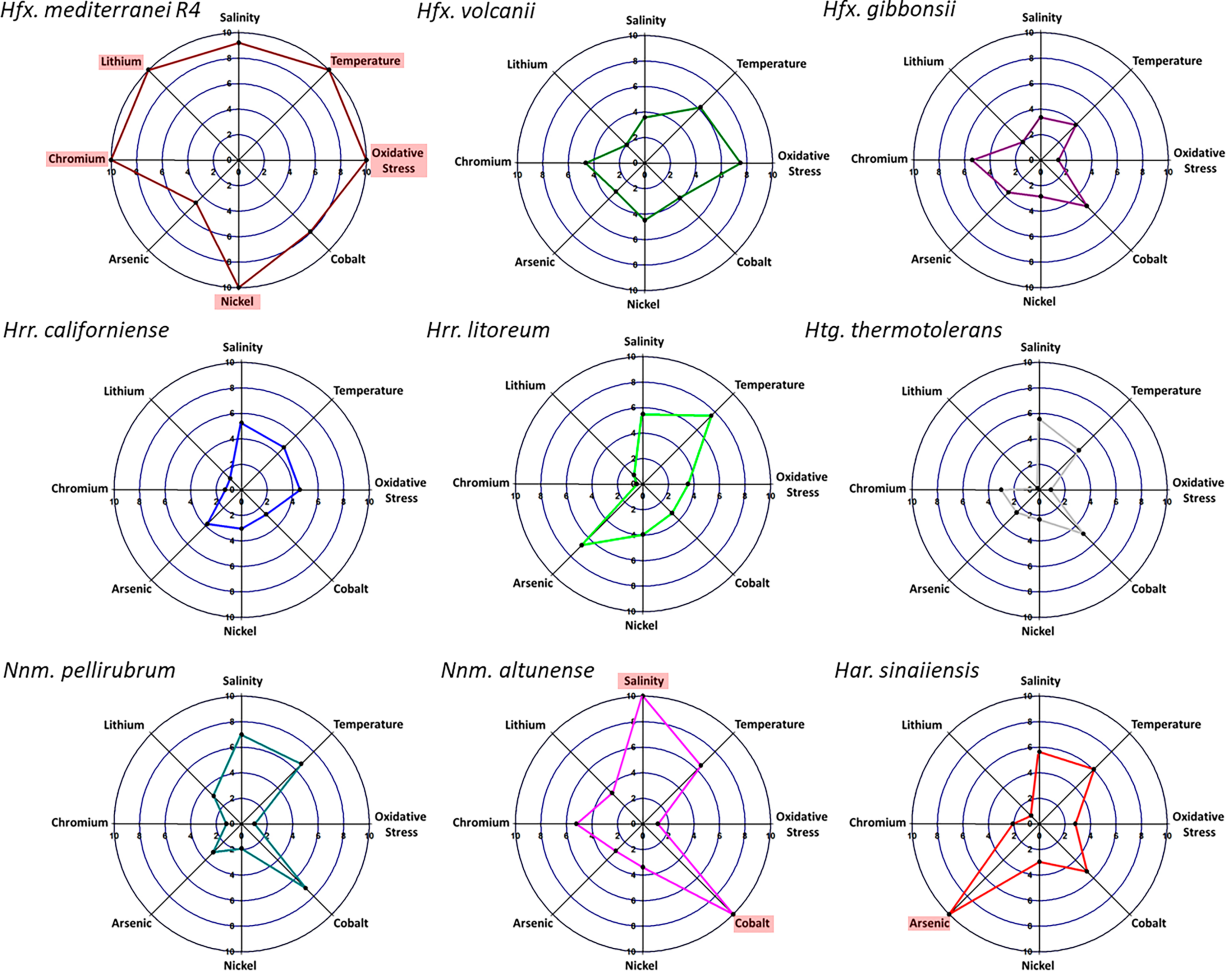
Polar plot illustrating the tolerance of each of the nine haloarchaea species towards the eight stress sources analysed. A score of 10 represents the maximum displayed tolerance. The coloured boxes highlight the maximum scores for each stressor.

After analysing the behaviour of these nine haloarchaea species under various stress sources, a polar plot has been constructed for each of them (Figure 7). Building upon all the aspects discussed in the previous sections, this mathematical descriptor tries to assign positive points in three directions: the higher the growth at a value within the range, the higher values within the range and a more significant number of values with observed growth within the range.

The results of this comprehensive analysis reveal that *Hfx. mediterranei* is the microorganism with the highest adaptability among the nine species analysed, obtaining the highest score in five of the eight proposed scenarios. Not only that, but it also achieves a high score in the remaining three scenarios. The only condition where it performs somewhat worse is in the presence of arsenic. It is essential to highlight that this species already exhibits a growth rate nearly double that of the others under the same control conditions, giving it an additional advantage. However, it also maintains a significant recalcitrance, being present in virtually all extreme concentrations for toxic substances, salinity and temperature. It seems highly probable that the distinct S‐layer profile, particularly its thickness and volume, as mentioned in the previous sections, is pivotal in facilitating its survival in harsh environments.

Nevertheless, further in‐depth molecular characterisation of this envelope is required to support this hypothesis. Two additional species have also displayed a fascinating tolerance profile. On the one hand, it appears that *Har. sinaiiensis* possesses a specialised cellular mechanism for arsenic detoxification, as it outperforms the others across the entire range of analysed concentrations (Figure 5B) and in this final assessment. At the same time, it does not exhibit any exceptional capability in the rest of the conditions.

On the other hand, *Nnm. altunense* exhibits the highest score in both salinity (although very close to *Hfx. mediterranei*) and in the presence of cobalt. Notably, while all species showed increased sensitivity to cobalt, this species displayed the highest resistance. Finally, it is worth noting that *Htg. thermotolerans* was the species with the lowest average score according to the mathematical descriptor. It is important to emphasise that this species exhibited the lowest growth rate, even under optimal control conditions. Even though it was one of the three species capable of demonstrating growth close to the control in the 70 different salinity and temperature conditions, its growth rate was substantially lower than the other species, significantly affecting its score. Notably, this species exhibited the poorest performance in lithium tolerance, with its growth completely inhibited by just 0.5 M of this cation.

Overall, this study significantly contributes to understanding stress responses in haloarchaea and highlights the relevance of these microorganisms in bioremediation efforts, particularly the potential use of *Haloferax mediterranei* and *Natrinema pellirubrum* in lithium recovery as an environmentally friendly biological methodology. These results represent a pioneering breakthrough, as this is the first study to assess the effects of multiple metals on nine different species of haloarchaea, thus opening new avenues of research in this emerging field. Future research will continue to explore the impact of lithium on these organisms, focusing specifically on lithium compounds such as lithium carbonate and silicate and their biosorption capacity. Additionally, further research will analyse the production of extracellular polymeric substances (EPS) under these conditions, as their production could play a key role in adapting microorganisms to metal stress and their potential application in bioremediation. This expanded approach will deepen our understanding of the molecular mechanisms underlying their tolerance and bioremediation capabilities.

## AUTHOR CONTRIBUTIONS

**Laura Matarredona:** Investigation; writing – original draft; writing – review and editing; methodology; conceptualization; formal analysis. **Basilio Zafrilla:** Writing – review and editing; formal analysis; data curation; methodology. **Mónica Camacho:** Resources; writing – review and editing; methodology. **María‐José Bonete:** Writing – review and editing; funding acquisition; project administration; conceptualization; supervision. **Julia Esclapez:** Conceptualization; funding acquisition; writing – review and editing; project administration; supervision; methodology; formal analysis.

## CONFLICT OF INTEREST STATEMENT

The authors declare no conflicts of interest.

## Supporting information


**Table S1.** Concentration value of the dissolved salts and the different ionic species in each of the salinity conditions tested.
**Table S2.** Growth rate and doubling time for haloarchaeal species under different salinity and temperature stress conditions.
**Table S3.** Detection of intracellular cation accumulation by inductively coupled plasma mass spectrometry (ICP‐MS) in haloarchaea species grown in the presence of LiCl.

## Data Availability

The data that supports the findings of this study are available in the supplementary material of this article.
