# Supplementary material for: Understanding the tolerance of halophilic archaea to stress landscapes
Source: Environ Microbiol Rep. 2024 Nov 20;16(6):e70039. doi: 10.1111/1758-2229.70039 (PMC11578932; doi:10.1111/1758-2229.70039)
Supplement: Supplementary file 1 — Table S1. Concentration value of the dissolved salts and the different ionic species in each of the salinity conditions tested. Table S2. Growth rate and doubling time for haloarchaeal species under different salinity and temperature stress conditions. Table S3. Detection of intracellular cation accumulation by inductively coupled plasma mass spectrometry (ICP‐MS) in haloarchaea species grown in the presence of LiCl. [file EMI4-16-e70039-s001.docx]

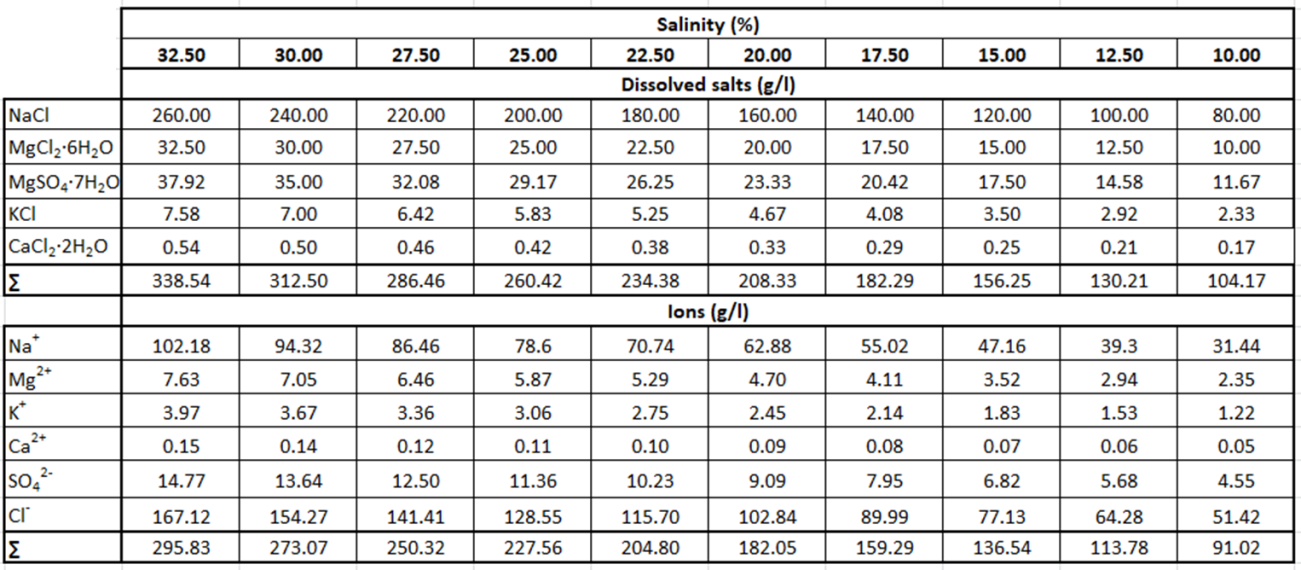
**Table S1:** Concentration value of the dissolved salts and the different ionic species in each of the salinity conditions tested.

**Table S2.** Growth rate and doubling time for haloarchaeal species under different salinity and temperature stress conditions.

| **Microorganism** | **Range of salinity (%) *** | **Range of temperature (ºC) *** | **Maximum growth rate (h^-1^) and doubling time (h)** | **Tolerance coefficient** |
| --- | --- | --- | --- | --- |
| *Hfx. mediterranei* R4 | 10-32.5  (20) | 32-52  (42) | 0.485 / 1.43 | 0.029 |
| *Hfx. gibbonsii* | 12.5-25  (17) | 37-52  (42) | 0.210 / 3.30 | 0.057 |
| *Hfx. volcanii* | 10-25  (15) | 37-52  (45) | 0.231 / 3.00 | 0.157 |
| *Hrr. californiense* | 15-30  (21) | 32-42  (37) | 0.215 /3.22 | 0.171 |
| *Hrr. litoreum* | 10-32.5  (20) | 32-52  (40) | 0.225 / 3.08 | 0.171 |
| *Nnm. pellirubrum* | 10-30  (20) | 32-45  (37) | 0.277 / 2.50 | 0.186 |
| *Nnm. altunense* | 10-32.5  (22.5) | 32-45  (37) | 0.250 /2.77 | 0.386 |
| *Htg. thermotolerans* | 10-32.5  (22.5) | 32-52  (37) | 0.116 /5.96 | 0.786 |
| *Har. sinaiiensis* | 15-30  (25) | 42-52  (45) | 0.232 / 3.00 | 0.171 |

* In parenthesis the optimum salinity and temperature for each species.

**Table S3.** Detection of intracellular cation accumulation by inductively coupled plasma mass spectrometry (ICP-MS) in haloarchaea species grown in the presence of LiCl.

|  | | **Na^+^**  (g/kg) | **K^+^**  (g/kg) | **Mg^2+^**  (g/kg) | **Ca^2+^**  (mg/kg) | **Li^+^**  (mg/kg) | **Total cations** (g/kg) |
| --- | --- | --- | --- | --- | --- | --- | --- |
| ***Hfx. mediterranei*** | **C** | 208 ± 5 | 47 ± 6 | 22 ± 6 | 143 ± 10 | 0.34 ± 0.02 | 278. ± 2 |
|  | **Li** | 226 ± 3 | 34 ± 1 | 21.4 ± 0.4 | 54 ± 3 | 266 ± 4 | 281 ± 9 |
| ***Hfx. volcanii*** | **C** | 134 ± 12 | 87 ± 12 | 14 ± 5 | 45 ± 12 | 2.6 ± 0.2 | 235 ± 32 |
|  | **Li** | 148 ± 48 | 7 ± 2 | 21 ± 4 | 263 ± 25 | 1076 ± 101 | 177 ± 53 |
| ***Hfx. gibbonsii*** | **C** | 170 ± 3 | 65 ± 11 | 16 ± 1 | 60.6 ± 0.8 | 0.93 ± 0.04 | 252 ± 13 |
|  | **Li** | 131 ± 1 | 132 ± 25 | 13.7 ± 0.3 | 43 ± 9 | 717 ± 75 | 278 ± 27 |
| ***Nnm. altunense*** | **C** | 105 ±7 | 109 ± 4 | 11 ± 2 | 62 ± 3 | 1.9 ± 0.1 | 226 ± 26 |
|  | **Li** | 75 ± 13 | 176.04 ± 0.02 | 10.1 ± 0.9 | 32 ± 3 | 715 ± 55 | 262 ± 14 |
| ***Nnm. pellirubrum*** | **C** | 116 ± 8 | 68 ± 9 | 18.6 ± 0.7 | 176 ± 8 | 2.62 ± 0.01 | 204 ± 15 |
|  | **Li** | 134 ± 12 | 37 ± 5 | 17.3 ± 0.7 | 124 ±14 | 1618 ± 739 | 191 ± 7 |
| ***Htg. thermotolerans*** | **C** | 177 ± 12 | 55 ± 12 | 18 ± 4 | 93 ± 12 | 1.2 ± 0.2 | 250 ± 50 |
|  | **Li** | 204 ± 51 | 53 ± 17 | 23 ± 6 | 99 ± 25 | 176 ± 36 | 280 ± 74 |

C: Control. Cells grown in basal medium

Li: Cells grown in basal medium with LiCl. *Hfx. mediterranei*, *Hfx. volcanii*, *Hfx. gibbonsii*, *Nnm. altunense* and *Htg. thermotolerans* were grown in the presence of 2M LiCl, and *Nnm. pellirubrum* with 0.5M LiCl.
